# Daphnetin: A bioactive natural coumarin with diverse therapeutic potentials

**DOI:** 10.3389/fphar.2022.993562

**Published:** 2022-09-29

**Authors:** Maira Javed, Ammara Saleem, Anne Xaveria, Muhammad Furqan Akhtar

**Affiliations:** ^1^ Department of Pharmacology, Faculty of Pharmaceutical Sciences, Government College University Faisalabad, Faisalabad, Pakistan; ^2^ Riphah Institute of Pharmaceutical Sciences, Riphah International University, Lahore Campus, Lahore, Pakistan

**Keywords:** daphnetin, neuroprotective, anti-inflammatory, anti-bacterial, psoriasis

## Abstract

Daphnetin (DAP), a coumarin derivative extracted from Daphne species, is biologically active phytochemical with copious bioactivities including anti-inflammatory, anti-oxidant, neuroprotective, analgesic, anti-pyretic, anti-malarial, anti-bacterial, anti-arthritic, neuroprotective, hepatoprotective, nephroprotective, and anti-cancer activities. A wide range of studies have been conducted exploring the significance and therapeutic potential of DAP. This study reviewed various databases such as NCBI, PubMed, Web of Science, Scopus and Google Scholar for published research articles regarding the sources, synthesis, and various bioactivities of DAP using different key words, including but not limited to “pharmacological activities,” “sources,” “neuroprotective effect,” “synthesis,” “cancer,” “anti-inflammatory effect” of “daphnetin.” Furthermore, this review encompasses both *in-vivo* and *in-vitro* studies on DAP for treating various diseases. A comprehensive review of the literature revealed that the DAP had a promising pharmacological and safety profile, and could be employed as a pharmaceutical moiety to treat a variety of illnesses including microbial infections, cancer, arthritis, hepatic damage, inflammation and neurological anomalies. The current review intends to provide an in-depth focus on all pharmacological activities and therapeutic approaches for the pharmaceutical and biomedical researchers.

## 1 Introduction

Phytochemicals are secondary metabolites that naturally exist in plants. These are categorized into various groups based on their chemical structure (Martinez et al., 2017). Coumarin is a naturally occurring secondary metabolite and benzopyrone derivative. This was one of the first metabolites to be identified in the 1930’s, and found in a variety of plant species (Archbold et al., 2011; Xu et al., 2011; Amin et al., 2014). A significant number of researches have been conducted to identify individual compounds, and develop procedures for their detection, synthesis, effectiveness and toxicity (Sovrlić and Manojlović, 2017). Since then, over 50 coumarins have been discovered in *Daphne* species. Depending on their structure, there are simple, dimeric or trimeric coumarins. *Daphne* contains various coumarins including: daphnetin, DAP-8-glucoside, daphnin, esculin, umbelliferone, and acetil-umbelliferon (Manojlović et al., 2012; Sovrlić et al., 2015). Rutarensin, daphnoretin, daphneretusin-A, dimethyl-daphnoretin-7-*O*-glucoside are categorized among dimeric coumarins while trimeric coumarin metabolites i.e., daphneretusin B, and triumbellin were also identified in *Daphne* species (Mansoor et al., 2013). This review elaborates sources, pharmacological activities as well as toxicity of daphnetin (DAP) so as to find and explore its therapeutic potential and promote the drug development.

DAP i.e., 7, 8-dihydroxycoumarin is generally an odorless and tasteless white or off-white powder that is freely soluble in ethanol, methanol and dimethyl-sulfoxide while slightly soluble in water (Shan et al., 2011). It has a molecular weight of 178.14 g/mole and melting point 262.0°C (Liao et al., 2013). It shows high solubility and permeability, and is metabolized by phase 1 reaction through CYP3A4 with a short half-life of 15 min. It exhibits poor bioavailability and absorbs through the intestine by passive diffusion. It is metabolized to methyl, glucuronide and sulfonate conjugated metabolites (Yang et al., 1999; Du et al., 2009; Shan, 2009; Liang et al., 2010; Chen, 2011; Zhang et al., 2014; Liang et al., 2015; Liang et al., 2017; Xia et al., 2018a). Some studies on DAP metabolism focused on glucuronidation, but other studies provided glimpse of other conjugated metabolites such as sulfonation, and methylation. In comparison to glucuronidation and sulfonation, the methylation pathway demonstrated a higher clearance rate (Liang et al., 2016).

**FIGURE 1:**
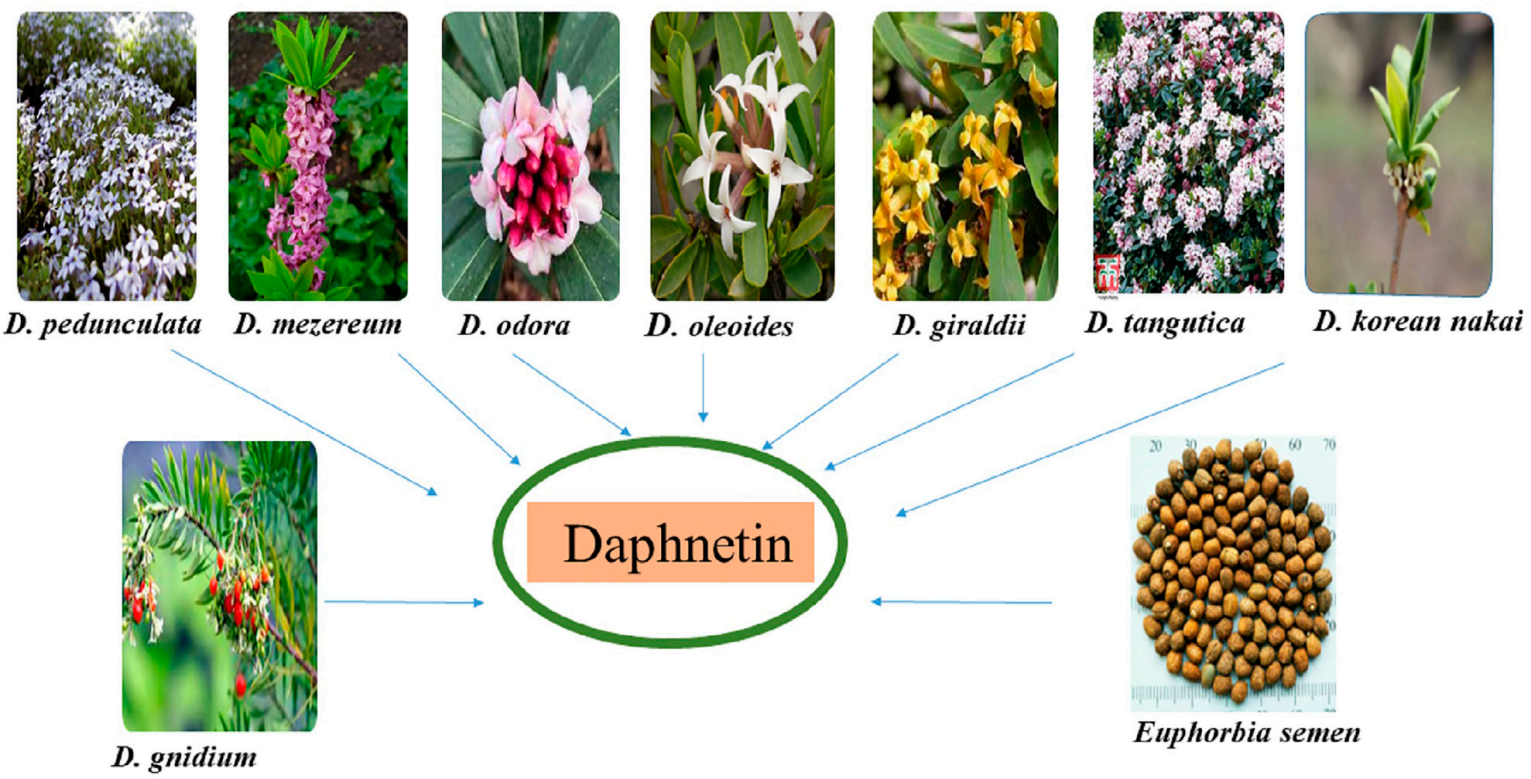
Different sources of daphnetin.

The DAP is derived from different *Daphne* species. *Daphne* is a genus comprising 70 to 95 species of perennial and evergreen shrubs of Thymelaeaceae family that is indigenous to India, Europe, and North Africa. These plants are renowned for their fragrant flowers and brilliantly colored fruit (Riveiro et al., 2010). DAP-8-glucoside is derived from *D. odora* in which it is formed from DAP-7-glucoside (Ueno and Saito, 1976; Halda et al., 1998). Other sources of DAP include *D. gnidium* (isolated from the leaves and stems), *D. mezereum* (synthesized from shoots), *D. giraldii, D. Koreana* Nakai*, D. tangutica* and *D. oleoides.* Seventeen compounds including DAP were isolated from *D. oleoides* (Brown, 1986; Riaz et al., 2016; Han et al., 2020; Khouchlaa et al., 2021)*. D. pedunculata* leaves and stems are also sources of DAP (Moshiashvili et al., 2020) as shown in Figure 1. *E. lathyris Linnaeus,* ethnically known as “*Euphorbia semen*” in East Asia, is also a source of coumarins including DAP. Previously, it was reported that simple coumarins including daphnetin, esculetin, esculin etc. Had been isolated and identified from the seeds of *E. semen* (Masamoto et al., 2003; Zhu et al., 2018). Different sources of DAP are shown in Figure 1.

This review focusses on the DAP-based treatment and prevention of diseases which are gradually receiving special attention due to underlying exceptional properties of DAP. In this context, an overview of DAP’s significance as an essential phytochemical and its intriguing uses have been presented and addressed. Relevant data were collected using various search engines such as Google scholar, PubMed, Web of Science, NCBI and Scopus by using the various search terms such as “daphnetin,” “Structure activity of daphnetin,” “sources of daphnetin,” “Synthesis of daphnetin,” “Traditional uses of *Daphne* species,” “isolation,” “physical properties,” “pharmacology of Daphentin” “hepatoprotective,” “neuroprotective,” “anti-inflammatory,” “anti-arthritic” and “anti-cancer” and “toxicity of daphnetin,” etc.

## 2 synthesis of Daphnetin

**FIGURE 2:**
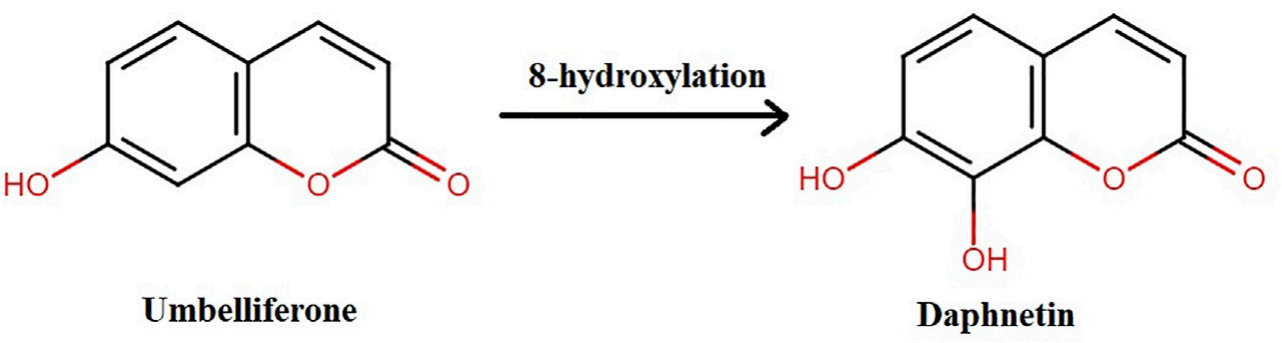
Synthesis of daphnetin.

DAP, naturally occurring or synthesized, has oxygen-containing heterocycles with a characteristic benzo-α-pyrone framework (Xia et al., 2018b). It is biosynthesized from shikimate pathway from L-Tyrosine and L-Phenylalanine (Norma Francenia, 2019). Previously, it was synthesized when pyrogallol and malic acid were heated in concentrated H_2_SO_4_ under nitrogen presence. It is also synthesized from umbelliferone by hydroxylation as shown in Figure 2 (NDong et al., 2003; Pan et al., 2017; Wang et al., 2020a).

## 3 Pharmacological activities of Daphnetin

**FIGURE 3:**
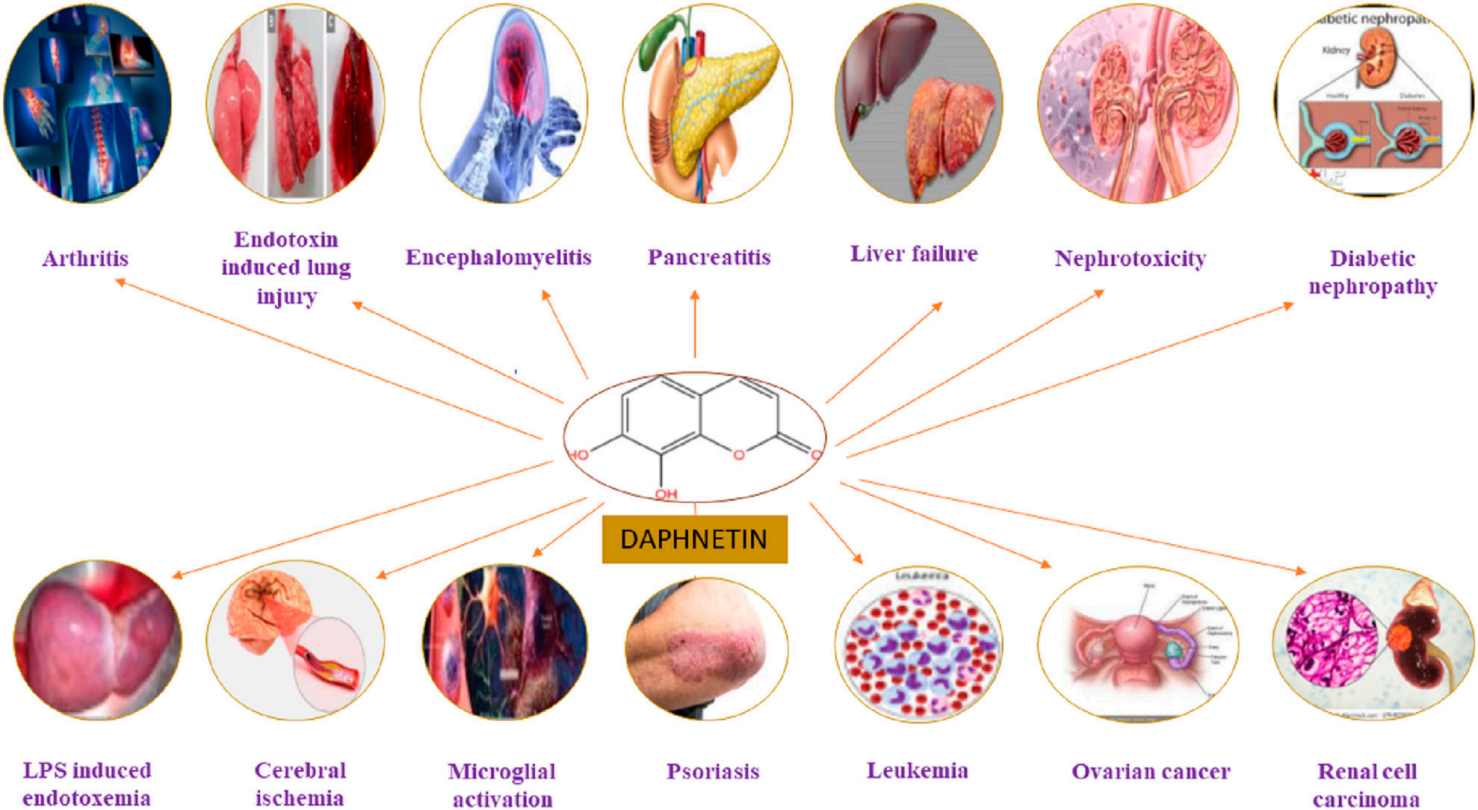
Pharmacological and therapeutic targets of Daphnetin.

The DAP has been used to treat coagulation disorders, various skin diseases, rheumatoid arthritis (RA), cancer, lumbago, and fever (Tu et al., 2012; Wang et al., 2013). It exhibited numerous pharmacological activities, including analgesic, anti-pyretic (Singh et al., 2021a), anti-arthritic, anti-inflammatory, anti-oxidant (Qi et al., 2016; Lv et al., 2018), anti-proliferative (Fylaktakidou et al., 2004; Kostova et al., 2011), anti-bacterial (Cottigli et al., 2001), neuroprotective (Qi et al., 2016), cardio-protective, nephroprotective, stroke, coagulation disorders, ischemic brain injury, hepatoprotective and anti-cancer activities (Pinto and Silva, 2017; Zhang et al., 2018; Boulebd and Khodja, 2021) as mentioned in Figure 3. It has been used for treating RA and coagulation disorders for long duration without significant toxic effects (Du et al., 2014). Pharmacological actions of DAP are summarized in Table 1.

**TABLE 1:** Pharmacological activities of Daphnetin.

Pharmacological action	*In-vivo/In -vitro* study	Cell line/Animal	Method	Dose	Molecular mechanism	Effects/Targets	References
Anti-stress	*In-vivo/in-vitro*	Kunming mice Cortical neurons from SD rat brains	Unpredictable stressor	2 and 8 mg/kg	↓GRs	↓ in spatial learning and memory improves the cognitive deficits caused by chronic stress	Liao et al. (2013)
Neuroprotective	*In-vivo*	E18 C57BI/6 mice	NMDA induced excitotoxicity	20 and 40 mg/kg	× NR2B-containing NMDA receptors	× apoptosis × calcium overload	Yang et al. (2014)
Hepatoprotective	*In-vivo In-vitro*	male C57BL/6 mice	Oxidative stress induced hepatotoxicity	20, 40 and 80 mg/kg	↑ Keap1-Nrf2/ARE-Trx-1↓ASK1/JNK, P53 protein	↓ t-BHP in HepG2 cells ↑ Nrf2/Trx-1 ↑GSH, ↓ ROS	Lv et al. (2020)
*Helicobacter Pylori* infection	*In-vitro*	20 H. pyloristrains isloted from gastric antrum		6.25 or 12.5 μg/ML	↑ DNA damage, ↑recA ×membrane changes	↓ bab A, urel transcription and H. pylori adhesion to GES-1 cell line	Wang et al. (2019)
Lung protection	*In-vitro/In-vivo*	Mice	Endotoxin induced Lung injury	5, 10 mg/kg	× activation of macrophage and human alveolar epithelial cells, induced TNFAIP3 ↓pro-inflammatory cytokines	NF-Kb related signal pathway, anti-inflammatory potential	Yu et al. (2014b)
	*In-vivo*	C57BL/6 mice	L-arginine	2-4 mg/kg i.p	↓IL-6, TNF-α, MPO ↓JAK-2, STAT-3	↓infiltration and cytokine secretion in inflammatory cells	Yang et al. (2021c)
Rheumatoid arthritis	*In-vivo*	Rats	Freund’s complete adjuvant induced arthritis	2.25 and 4.5 mg/kg	↓IL-1, TNF-α and MIF	↓paw swelling and arthritic scores × inflammatory cells infiltration and articular cartilage degeneration	(Gao et al., 2008; Yao et al., 2011; Tu et al., 2012)
	*In-vivo*	Female Wistar rats	Collagen induced arthritis	1 and 4 mg/kg	↑ Foxp3	↓Th1/Th2/Th17	Yao et al. (2011)
					↓ activity of Th17 ↓ RORγt, NF-KB, CD77 ↓IL-10 ↑ Tregs	↓ paw swelling ×hyperplasia of synovial, destruction and degeneration of chondrocytes Modulate balance of Th17 and Tregs	
Osteoporosis	*In-vivo In-vitro*	Sprague Dawley rats MC3T_3-_E_1_ pre osteoblasts	Dexamethasone induced	1 and 4 mg/kg	activate Wnt/GSK-3β/*ß* catenin signaling pathway	↓ body weight gain, bone mineral content and microstructure parameters	Wang et al. (2020b)
						↑ osteoblast proliferation, differentiation and mineralization	
Hepatoprotective	*In-vivo*	Mice	Lipopolysaccharide/D-galactosamine induced liver failure	20, 40, 80 mg/kg	× Inos × COX-2 ↑ autophagy ↑ pro-autophagy protein expression	↓ ALT, AST ↓pro-inflammatory cytokines ↓MDA ↓myeloperoxidase ↑GSH, SOD level × MAPK, NF-kβ, NLRP3	Lv et al. (2018)
Liver cancer	*In-vivo*	Huh7 and SK-HEP-1		0, 5, 10, 50 and 100 µM	↑ G1 phase arrest	×cell viability	Liu et al. (2022)
						×tumorigenesis	
						↑cell apoptosis	
						×Wnt/*ß* catenin signaling	
Nephroprotective	*In-vivo*	C57BL/6 mice	Cisplatin induced nephrotoxicity	2.5,5,10 mg/kg	↓ TNF- α, IL-1β, ROS, MDA	×NF-kB signaling pathway activate Nrf2 pathway	Zhang et al. (2018)
						↓ BUN, creatinine	
						↓ renal injury	
						↓inflammation, oxidative stress, apoptosis	
	*In-vivo*	WT and Nrf2 mice	Cisplatin induced nephrotoxicity	20-40 mg/kg	↑SOD, GSH, SIRT1, SIRT6, HO-1, Nrf2, NQO1 ↓MDA, MPO	↓ BUN, creatinine ↓ renal injury ↓inflammation, oxidative stress, apoptosis	Fan et al. (2020)
Diabetic nephropathy	*In-vivo*	mesangial cells	High Glucose induced	0, 10, 20, 40 μM	↑ Nrf2 ×p-Akt ×p-p65	↓ ROS, MDA ↓TNF- α, IL-1β ↓IL-6, ↓fibronectin ↓collagen IV ↑ SOD activity ↓ cell proliferation	Xu et al. (2019)
Cerebral Ischemia/Reperfusion injury	*In -vivo*	C57BL/6mice	Middle cerebral artery occlusion	5, 10, 20 mg/kg	↓TNF- α, IL-1β, IL-6, TLR4	×TLR4/NF-kβ ↓IkBα degradation ↓neural cell apoptosis	Liu et al. (2016a)
	*In-vitro*	Hippocampal neuron	Reoxygenation induced lung injury	10, 20 and 40 µm	↑ Nrf2 ↑ HO1	× oxidative stress and neuronal apoptosis	Zhi et al. (2019)
Ischemic brain injury	*In-vitro*	HT22 cells	glutamate induced toxicity in hippocampal HT22 cell	5, 10, 25, 50, 75, and 100 μM/L	×NF-kB pathway	↑SOD, GSH ×TLR4/NF-kB pathway	Du et al. (2014)
Microglial activation	*In-vivo*	Murine microglia	Intracellular signal transduction	0–160 µm	×iNOS ×COX-2	↓ TNF-α, IL-1β, IL-6 NO, ×microglial activation ↓Th17 development ×NF-kB × MAPK × IKK /IkB PI-3K/Akt	Yu et al. (2014a)
Psoriasis	*In-vitroIn-vitro*	HaCaT human keratinocytes in Mice	imiquimod induced psoriasis like skin lesion	50-100 mg/ cm	↓ IL-1β, IL-6, IL-8, IL-17A, TNF- α, IL23A, MCP-1	pathway ×p65 phosphorylation ×nuclear translocation ↓erythema ↓scaling, epidermal hyperplasia, inflammatory cells infiltration	Gao et al. (2020)
Cell proliferation and Estrogenicity	*In-vivo In-vitro*	MCF-7 cells Female mice		17.5, 35, 70, 140 mg/kg	↓ Cyclin D1 ↑ p27	↑GO phase ↓G1 phase ↑S phase ↑G2 phase ↑M phase ↓cyclin/CDK2 ↓cyclin D1/CDK4 ×cyclin D1	Jiménez-Orozco et al. (2011)
Leukemia	*In-vivo*	Albino Wistar rats	Benzene induced	12.5, 25, 50 mg/kg	↑sphingosine1-phosphate receptor-1 ↓SGOT ↓Cytochrome P450 ↓CYP2E1	↓ NF-kB ↑Hematological parameter ↑nucleated bone marrow cells ↑megakaryocyte , SOD, GSH ↓MDA, ↓8- OhdG ↑albumin, total protein ↓BUN, bilirubin ↓prothrombin time	Pei et al. (2021)
Ovarian cancer	*In-vitro*	A2780		5, 10, 20, 40 μg/ml	↓ p-Akt ↓ p-mTOR ↑p-AMPK, LC3 II, p62	↑ROS production ×cell proliferation ↑apoptosis, autophagy, Blood count, Hemoglobin↓ proinflammatory cytokines	Fan et al. (2021a)
Human renal cell carcinoma	*In-vitro*	A-498 cells		10 and 50 µm	↑p38 MAP kinase ↑cytokeratin 8 and 18	MAPK Signaling pathway ×ERK1/ERK2 pathway ×S phase transition ×DNA synthesis	Finn et al. (2004)
Corneal inflammation and neovascularization	*In-vivo*	Male ICR mice	Alkali burn	(10-20 μmol/L) DAP eyedrops, q.i.d	↓HUVECs ↑STAT3, ERK, AKT	×corneal inflammation (↑VEGF-A) and neovascularization (↑TLR4/NLRP3)	Yang et al. (2022)
In various tumors	*In-vivo*	Female in bred BDF1 C57Bl/6	S180 sarcoma, MXT breast adenocarcino ma, C26 colon carcinoma	10,20 and 40 mg/kg	↑p38 MAP kinase ↑cytokeratin 8 and 18 ↑pro-apoptotic caspase-3	×mitogenic pathway ↓Cyclin D1 ×S phase ×Akt/ NF-kβ pathway ×proliferation	Jiménez-Orozco et al. (2020)
	*In-vivo*	Murine	Osteosarcoma LM8 cells	30-60 µm	↓ RhoA ↓Cdc_42_	↓intracellular stress fibers and filopodia	Fukuda et al. (2016)
Mitochondrial dysfunction and cell death	*In-vitro*	C57Bl/6 mice	Tert-butyl hydroperoxide	2.5,5,10 µg/ml	↑HO-1, SOD ↑NADPH, NQO1, GCLM ↑GCLC, BCl2 ↓ Bax, Caspase 3	×ROS production ×cytochrome c release, NLRP3 activation ↑Nrf2 pathway activate JNK and ERK	Lv et al. (2017)
CFA induced inflammatory pain	*In-vivo*	Murine	CFA	4 and 8 mg/kg	↓ spinal pro-inflammatory cytokines	×spinal glial activation × NF-kβ pathway ↑Nrf2 pathway/HO-1 signaling pathway	Yang et al. (2021a)
Inflammatory bowel disease	*In-vivo*	Mice	Fecal transplantation	16, 8, 4 mg/kg		↑ T reg cells development ↓ Th 17 cell differentiation	Ji et al. (2019)
Lipid metabolism	*In-vitro*	HepG2 cells		5, 20 and 50 µm	↑ PNPLA3	↓TG	Liu et al. (2019)
Insulin resistance	*In-vitro*	HepG2 cells		20 and 50 µm	↑ pAKT/AKT P13K	↑ glucose uptake	Liu et al. (2019)
Oxidative stress	*In-vitro*	HepG2 cells		5, 20, and 50 µm	↓ CYP2E1 and CYP4A ↑ Nrf2	↓ oxidative stress	Liu et al. (2019)
Angiogenesis	*In-vivo*	Rat	TNF and VEGF induced	9.375–900 µM	↓ c-Src, FAK, ERK1/2, Akt, VEGFR2, iNOS, MMP2	× angiogenesis ×migration ×invasion ×tube formation × NF-kβ pathway ×TNF-α induced IkBα degradation ×translocation of the NF- kβp65 protein ↑apoptosis	Kumar et al. (2016b)

Abbreviations: Inhibits; ↑: Upregulates, Increase; ↓: Downregulates, Decrease; CUS, chronic unpredictable stress; GRs: Glucocorticoid receptors; ALF, acute liver failure; APAP, acetaminophen; ASK1, Apoptosis signaling-regulating kinase 1; AREs, Antioxidant response elements; HO-1, Heme oxygenase-1; JNK, c-Jun N-terminal kinase; NF-κB, Nuclear factor-kappaB; Nrf2, Nuclear factor erythroid 2-related factor; 2NLRP3, Nucleotide-binding domain-like receptor protein 3 ROS, reactive oxygen species; Trx-1, Thioredoxin-1; Txnip, Thioredoxin-interacting protein; PALI, pancreatic acute lung injury; JAK-2, Janus kinase-2; STAT-3, Signal transducer and activator of transcription 3; VEGFR2, Vascular endothelial growth factor 2; iNOS, inducible nitric oxide synthase.

### 3.1 Neuroprotective action

Nerve cells interact with one another to carry out various physiological functions. Any communication breakdown in the brain, even in a solitary area, can impair the operation of other brain regions and is the main factor in catastrophic neurological illnesses or neurodegenerative disorders of the central and peripheral nervous systems. Progressive neuronal degeneration causes temporary or permanent sensory loss in a variety of neurodegenerative disorders. The reactive oxygen species (ROS) and inflammatory signaling molecules i.e., tumor necrosis factor alpha (TNF-α), and interleukin (IL)-6 are the key contributors to neurodegenerative disorders (Qi et al., 2016; Chitnis and Weiner, 2017; Boulebd and Khodja, 2021). For neuroprotective action, DAP reduces Toll-like receptor-4 (TLR-4), nuclear factor-ĸβ (NF-ĸβ), and other pro-inflammatory cytokines. It also inhibits JAK/STAT phosphorylation which is responsible for the increase of pro-inflammatory cytokines and enzymes, culminating in the reduction in COX-2 and inducible nitric oxide synthase (iNOS) levels. It significantly enhances the Nrf-2 expression. DAP is reported to enhance Heat shock protein (HSP)-70 by downregulating the expression of NF-ĸβ and mitogen-activated protein kinase (MAPK) at the molecular level, causing the enzymes to regulate neuronal apoptosis by increasing or decreasing the phosphorylation of pro-apoptotic proteins (Bax/Bad) and an anti-apoptotic protein (Bcl-2) (Singh et al., 2021a).

#### 3.1.1 Cognition and memory

DAP has shown significant potential to prevent memory loss and cognition. It inhibited apoptosis and calcium overload induced by down-regulating NR2B-containing N-methyl-D-aspartate (NMDA) receptors as well as calcium accumulation which was activated by glutamate and caused excitation of neurons. DAP exhibited neuroprotective properties by preventing NMDA-induced neuronal cell loss and regulating the balance of Bcl-2 and Bax expression in cortisol neurons of mice (Yang et al., 2014).

The neuroprotective effect of DAP has been reported in the posterior cerebral artery occlusion (MCAO)/reperfusion mice model. The DAP (1 mg/kg) showed a substantial reduction in cerebral infarct volume (Du et al., 2014). It has a neuroprotective effect in stressed mice on microglial activation and its subsequent inflammatory response. The pre-incubation with DAP dramatically inhibited TNF-α and IL-1 production in lipopolysaccharide (LPS) or *ß*-amyloid activated BV2 cells.

**FIGURE 4:**
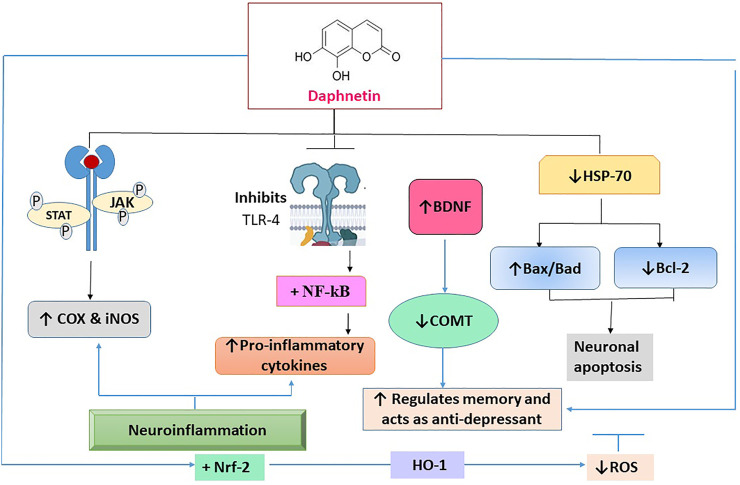
The molecular interaction of multiple mediators implicated in the neuroprotective effect of daphnetin. TLR-4: toll-like receptor -4; NF-ĸβ: nuclear factor-ĸβ; Janus kinase: JAK/STAT; COX-2: cyclooxygenase -2; iNOS: inducible nitric oxide; Nrf-2: Nuclear factor erythroid 2-related factor; MAPK: mitogen activated protein kinase; Bcl-2: pro-apoptotic proteins; Bax/Bad; anti-apoptotic protein; ROS: reactive oxygen species.

The MAPK and protein kinase B (Akt) pathways play a negative role in the anti-inflammatory action of DAP. Furthermore, pre-treatment with Wortmannin, a PI-3 k/Akt inhibitor, resulted in a significant decrease in LPS-induced TNF-α and nitric oxide generation in BV2 cells, demonstrating an opposing role of the MAPK/Akt pathway in mediating anti-inflammatory effect of DAP. It also reduced the expression of NF-κB to promote neuroprotection (Yu et al., 2014a). The neuroprotective action of DAP is presented in Figure 4.

In another study, the neuroprotective effect in mice was studied by utilizing the middle cerebral artery occlusion (MCAO)/R model. It reduced the extent of the MCAO/R-induced cerebral infarct, neuronal apoptosis, and brain IL-1β, IL-6, TLR-4/NF-kB, and TNF-α levels in the cerebral cortex (Vivier et al., 2016). Lei Shen and his coworkers showed that DAP had reduced endotoxin lethality in a mouse model of LPS-induced endotoxemia and suppressed the inflammatory response to LPS in Raw264.7 cells by inhibiting ROS generation, JAK1 and JAK2, and enhancing suppression of STAT1 and STAT3 phosphorylation, and ultimately prevented the STAT1 and STAT3 transport in the nucleus (Shen et al., 2017).

Several preclinical researches have revealed that DAP had improved spatial memory and depressed behavior. The chronic unexpected stress mice model was used to assess the effect of DAP. It improved the Chronic Unpredictable Stress (CUS) impaired spatial memory as indicated by mouse performance in the Morris water maze test. DAP (2 and 8 mg/kg) injection reduced the immobilization time in a forced swim test compared to the CUS-treated group, confirming the involvement of DAP in improving spatial memory and restoring depressive behavior in mice (Liao et al., 2013).

#### 3.1.2 Stroke

Cerebral ischemia is caused by impaired blood flow and is accompanied by an inflammatory reaction, release of cytokines, and inflammatory mediators that play a pivotal role in the development of stroke (Iadecola and Alexander, 2001). The TLR4 is highly induced after reperfusion injury. In an earlier study, DAP was used for treating cerebral ischemia/reperfusion injury and it was found to exhibit neuroprotective and anti-inflammatory effect by inhibiting TLR4/NF-kβ pathway, alleviating the production of inflammatory cytokines and neural cell apoptosis (Liu et al., 2016a). DAP (at 5, 10, 25, 50, 75, and 100 μM/L) displayed dose-dependent neuroprotective action in glutamate-induced toxicity in hippocampal HT22 cells and ischemic brain injury by restoring reduced glutathione (GSH) and superoxide dismutase (SOD) (Du et al., 2014).

It was further found that DAP suppressed oxidative stress and cell apoptosis in hippocampal neurons. It increased nuclear translocation of nuclear factor erythroid 2 (Nrf2) and HO-1 expression in neurons exposed to reoxygenation-induced cell injury at 10, 20, and 40 µM doses. DAP inhibited oxidative stress and neuronal apoptosis by activating Nrf2/HO-1 signaling pathway (Zhi et al., 2019).

### 3.2 Hepatoprotective action

Excessive generation of ROS causes significant damage to cell membrane phospholipids, proteins and DNA leading to a variety of disorders (Blair, 2008; Mendes-Braz and Martins, 2018; Zhao et al., 2018). DAP reduced hepatotoxicity caused by tert-butyl hydroperoxide (t-BHP) and acetaminophen through the modulating Nrf2/Trx-1 pathway. It effectively prevented t-BHP-induced hepatotoxicity by regulating Nrf2/Trx-1 pathway in HepG2 cells. Moreover, it inhibited ASK1/JNK activation and decreased the acute liver failure (ALF), cytochrome C, and Bax mitochondrial translocation, all of which concomitantly restored the mitochondrial function. It is also found that DAP inhibited inflammatory reactions in the liver by inactivating the thioredoxin-interacting protein (Txnip)/NLRP3 inflammasome. It also improved the Nrf2 nuclear translocation and Trx-1 expression (Lv et al., 2020). It inhibited MAPK, NF-kβ, nucleotide binding domain like receptor protein 3 (NLRP3) and decreased the pro-inflammatory cytokines in acute liver failure (ALF) (Lv et al., 2018). The hepatoprotective mechanism of DAP is given in Figure 4.

A study reported that the DAP had reduced the lipid accumulation within the hepatocytes by regulating PI3K expression and pAKT/AKT levels. Moreover, it decreased the insulin resistance by promoting the hepatocellular glucose uptake through upregulating the expression of Nrf2. In addition, DAP reduced the level of ROS in hepatocytes by downregulating the expression of CYP2E1 and CYP4A (Liu et al., 2019). In an earlier study, DAP improved carbon tetrachloride (CCL4)-induced biochemical changes. It decreased the CCL4 induced-lipid peroxidation and boosted the antioxidant defense system. DAP induced nuclear translocation of Nrf2 related factor 2 to induce the expression of hydroxyl ion. Thus, DAP prevented the hepatotoxicity induced by oxidative stress by activating Nrf2-mediated hydroxyl ion expression (Mohamed et al., 2014).

### 3.3 Effect on heavy metal and endotoxin induced lung injury

It is found that DAP exhibited a phenomenal anti-oxidant in arsenic-induced cytotoxicity in human lung epithelial cells. DAP (at 2.5, 5, 10 g/ml) progressively shielded Beas-2B cells from NaAsO2-induced apoptosis as well as arsenic cytotoxicity *via* Nrf2-dependent pathway and increased GSH level (Lv et al., 2019).

Endotoxin is an important toxin precipitating lung injury and is responsible for increased serum concentration of all cytokines and growth factors (Rojas et al., 2005). DAP at 5 and 10 mg/kg provided considerable protection from endotoxin-induced acute lung injury in mice by inhibiting the activation of macrophages and human alveolar epithelial cells through reducing the production of inflammatory mediators, induction of TNF-α induced protein 3 (TNFAIP3) and decreasing the expression of iNOS and NF-κB to attenuate inflammation. It also downregulated the phosphorylation of MAPKs including p38, extracellular signal regulated kinase (ERK), and JNK kinases (Yu et al., 2014b).

### 3.5 Anti-bacterial action

DAP was investigated for anti-bacterial activity against *Helicobacter pylori*; a Gram-negative bacterium that usually colonizes stomach causing gastritis and peptic ulcers (Zali, 2011). DAP showed virtuous activity against multidrug resistant (MDR) *H. pylori via* enhancing DNA damage, phosphatidylserine (PS) translocation, and recA expression while downregulating blood group antigen binding adhesion (babA) and urease l (urel) with the decrease in the attachment of *H. pylori* to GES-1 cells with minimal inhibitory concentration (MIC) from 25 to 100 μg/ml (Wang et al., 2019); (Walker and Crabtree, 1998). It also showed antibacterial activity by destroying cell wall and preventing membrane coherence of *Pseudomonas fluorescens* and *Shewanella putrefaciens* with MIC of 0.16 and 0.08 mg/ml respectively (Liu et al., 2020). In another study, the effect of DAP on the *Ralstonia solanacearum* was investigated in which it was found that DAP had exhibited strongest anti-bacterial effect due to the presence of hydroxylation at C6, C7, or C8 which increased its anti-bacterial effect by destructing the bio-membrane against *R. solanacearum* with the MIC at 64 mg/L (Yang et al., 2016).

DAP (at 10 mg/kg i.p.) was used to treat bacterial pneumonia caused by methicillin-resistant *Staphylococcus aureus* (MRSA) in C57BL/6 mice. It protected against inflammation, tissue damage, and stimulated the mTOR-dependent autophagy pathway, which resulted in the increased bactericidal activity of macrophages by suppressing ROS production (Zhang et al., 2019).

### 3.6 Anti-malarial action

Malaria is one of the major fatal diseases, affecting around 1 million people worldwide and leading to death (Hu et al., 2018). DAP and its two derivatives, DAP78 and DAP79, have demonstrated anti-malarial activity against *Plasmodium falciparum*; nevertheless, DAP functions as an iron chelator, and its anti-malarial potency decreased significantly with time, leading its chelating action to be abolished (Huang et al., 2006). In another investigation, DAP was found to have a high iron chelating activity when compared to the potent iron-chelator desferroxamine B at different dosages (Mu et al., 2002). It caused 50% inhibition of ^3^H-hypoxanthine incorporation by *P. falciparum* at 25 and 40 µM. DAP did not immediately generate superoxide under *in-vitro* conditions, therefore it is not considered an oxidant. However, during *in-vivo* studies, it significantly prolonged the survival of mice infected with *P. yoelli* (Yang et al., 1992). Wang et al. (2000) reported the schizontocidal activity of DAP by using *P. falciparum* FCC1 strain *in-vitro*. The *in-vivo* activity was evaluated against *P. berghei* in Anka mice at the dosage of 10–100 mg/kg/day which demonstrated positive outcome.

### 3.7 Anti-inflammatory and anti-arthritic actions

The excess endogenous production of ROS leads to oxidative stress due to decreased concentration of GSH, SOD, and increased level of malondialdehyde (MDA). Oxidative stress also causes activation of the NF-κB pathway. This pathway controls the release of different cytokines by directing the expression of number of pro-inflammatory cytokines, inhibiting the apoptosis proteins (IAPS) and COX-2 which leads to inflammation. For anti-inflammatory action, DAP inhibits these pathways.

Adjuvant-induced arthritis is an autoimmune disorder characterized by chronic inflammation of joints that exhibits the same pathological response as that of RA (Connor et al., 1995). Various pro-inflammatory mediators play a significant role in the pathogenesis of this disorder (Barsante et al., 2005). DAP significantly attenuated the poly-arthritis by suppressing the production of pro-inflammatory cytokines (IL-1 and TNF-α) (Gao et al., 2008).

In another study, DAP alleviated the inflammation and pathological changes in the joint tissue, synovial hyperplasia, and chondrocyte degeneration in collagen-induced arthritis (CIA) in female rats at 1 and 4 mg/kg by restoring the expression of Th1/Th2/Th17 type cytokines, Foxp3, IL-17, IL-6, TGF- β, IL-4, and IFN- γ (Tu et al., 2012). In another study, it inhibited the proliferation of fibroblast-like synoviocytes (FLS) in rats with CIA and induced apoptosis by suppressing PI3k/AKT/MTOR signaling pathway at 0-60 μg/ml (Deng et al., 2020). In another study, DAP was combined with B cell lymphoma 2 targeted small interfering RNA (si-Bcl2) on fibroblast-like synoviocytes (FLS) in rats with CIA by downregulation of Bcl2. When si-Bcl2 was combined with DAP (40 μg/ml), it increased the effect *via* promoting apoptosis on RAFLS and by reducing the expression of Bcl2 and STAT3 (Chen et al., 2018).

In a previous study, DAP reduced the serum level of Th17, Th2, and Th1 type cells and upregulated the levels of Tregs in arthritis rats at 1 and 4 mg/kg. It also decreased ROR*γt,* NF-kB, and CD77 in joint tissue while increased the expression of Foxp_3_ and IL-10. Thus it modulated the balance of Tregs and Th_17_ cells and is considered to be an effective agent in the treatment of CIA in rats (Yao et al., 2011).

Zhang et al. (2020) reported the chondroprotective effect of DAP against osteoarthritis. DAP (at 12, 24 and 48 ng/ml) profoundly protected chondrocytes of rabbits by averting IL-1β, -6,-12, MMP3,-9, and -13 and decreasing the caspase-3 and BAX while increasing BCL-2. In another study, DAP exhibited anti-arthritic action by demethylation of pro-apoptotic genes in synovial cells (FasL and P53). For this purpose, MTT analysis was performed on CIA-treated rat synovial cells to determine the inhibitory effect of DAP and DNA methyltransferase inhibitor drug (5-aza-dc) in the range of 1.25-40 μg/ml. It inhibited cell growth in synovial cells in a dose and time dependent manner (Shu et al., 2014). Zheng et al. reported anti-arthritic action of DAP using collagen induced FLS. It escalated caspase 3, 8, and 9, Bax, FasL, and cytochrome c (Cyt-c) with the reduction in Bcl-2 and enhanced the Cyt-c discharge from mitochondria to the cytosol (Zheng et al., 2020). In another study, DAP at 4 and 8 mg/kg inhibited spinal glial activation in murine mice provoked by CFA. It also decreased the expression of pro-inflammatory cytokines. It inhibited the NF-κβ pathway and activated the Nrf2/HO-_1_ signaling pathway (Yang et al., 2021a).

### 3.8 Osteoporosis

Glucocorticoids are effective agents in treating inflammatory and autoimmune diseases. While its long-term usage results in osteoporosis. DAP at 1 and 4 mg/kg exhibited the therapeutic action against dexamethasone induced osteoporosis in male rats by restoring bone mineral content, microstructure parameters, and bone turnover. *In-vitro*, it promoted osteoblast proliferation, differentiation, and mineralization in pre-osteoblasts by activating Wnt/GSK-3β/β catenin signaling pathway (Wang et al., 2020b).

### 3.9 Multiple sclerosis

Multiple sclerosis (MS) is an inflammatory and neurodegenerative illness that is identified by projected inflammation, axonal injury, and demyelination. DAP (8 mg/kg) has exhibited an immune-regulatory role in autoimmune encephalomyelitis in the murine model used for MS (Leung et al., 2011). Autoimmune encephalomyelitis is a demyelinating inflammatory illness of the central nervous system caused in experimental animals by an immune response to myelin epitopes (Fletcher et al., 2010). T cells attract macrophages, microglia, and astrocytes which release inflammatory mediators such as nitric oxide (NO), and ROS. DAP therapy lowered the level of pro-inflammatory cytokines, induced heme oxygenase-1 (HO-1), decreased the level of MDA, and displayed anti-inflammatory and neuroprotective effects in mice at 8 mg/kg (Wang et al., 2020c). In a previous study, DAP administered for 28 days mitigated the encephalomyelitis in mice *via* suppressing the activation, maturation, and antigen-presenting capability of Dendritic cells, and regulated NF-κB signaling (Wang et al., 2016).

### 3.10 Systemic lupus erythematosus

Li et al. reported the anti-inflammatory potential of DAP in the NZB/WF1 systemic lupus erythematosus (SLE) murine model. In the SLE-prone NZB/W F1 mice, DAP (at 5 mg/kg) treatment enhanced the survival rates, reduced renal damage and blood urea nitrogen levels, and lowered the serum autoantibody production. Furthermore, its therapy significantly reduced the serum levels of TNF-α and IL-6, inhibited NF-kB activity, lowered the nuclear factor of activated T-cell protein production, and increased the A20 protein expression in SLE-prone NZB/W F1 mice. Finally, DAP reduced the inflammation in the NZB/WF1 murine SLE model *via* NF-κB suppression mediated by A20 overexpression (Li et al., 2017).

### 3.11 Anti-psoriasis action

Psoriasis is a chronic inflammatory disease of the skin characterized by excessive proliferation, abnormal differentiation of keratinocytes, and infiltration of inflammatory cells into the *epidermis* and dermis. Hyperproliferation of keratinocytes and extreme inflammatory response play a pivotal role in its pathogenesis. Cytokines secreted by immune cells cause keratinocytes’ hyperproliferation which produces pro-inflammatory cytokines to potentiate inflammatory response. A previous study showed the anti-psoriatic activity of DAP in HaCaT keratinocytes mouse which occurred through the downregulation of inflammatory cytokines and suppression of NF-κB signaling pathway (Gao et al., 2020). DAP also decreased the epidermal hyperplasia and infiltration of inflammatory cells in imiquimod induced skin lesions in mice. In another research, DAP above 40 μM caused a decrease in cell viability in human HaCaT keratinocytes by upregulation of IL-1, -6, -8, TNF-α, and IL-23A while inhibiting P65 phosphorylation and nuclear translocation. Additionally, it improved the inflammation, erythema, scaling, and epidermal thickness of psoriatic mice (Gao et al., 2020).

### 3.12 Anticancer action

DAP is known for anticancer potential against leukemia, ovary, kidney, colon, and liver cancers. Uncontrolled proliferation and suppression of apoptosis lead to cancer. Mitogen pathways are responsible for regulating apoptosis. DAP is a protein kinase inhibitor; therefore, it significantly suppresses this pathway and acts as an anti-proliferative agent. It also acts at different phases of the cell cycle. DAP inactivates Akt/NF-κB (an anti-apoptotic pathway), JNK, MAPK, and ERK pathways that are responsible for causing cancer. DAP activated Keap1-Nrf2 pathway that protected the cell against oxidative stress by activating transcription of several cytoprotective genes thus helping to combat cancer (Figure 4) (Jiménez-Orozco et al., 2020). In a previous study, effect of DAP (at 2.5, 5, and 10 μg/ml) on tert-butyl hydroperoxide (t-BHP) induced mitochondrial dysfunction and cell death in C57B1/6 mice and RAW 264.7 cells revealed that the DAP suppressed the production of ROS by stimulating various anti-oxidant genes and activating Nrf2 pathway that protected the body against oxidative damage. Activation of Nrf2 pathway suppressed NLRP3 activation, thus inhibiting the activation of caspases and release of pro-inflammatory cytokines. In this way, DAP protected the body from cell death and mitochondrial dysfunction (Lv et al., 2017).

In another study, anti-proliferative properties of DAP in cancer cells were reported. DAP inhibited migration and invasion of highly metastatic murine osteosarcoma LM8 cells. It reduced the intracellular stress fibers and filopodia. It also decreased the expressions of RhoA and Cdc_42_ (Fukuda et al., 2016).

#### 3.12.1 Kidney cancer

The human renal cell carcinoma (RCC) accounts for up to 90% of kidney cancers due to the alterations of the genes responsible for controlling cell division (Motzer et al., 1996; Hsieh et al., 2017). MAPKs pathway causes activation of transcription factors which in turn regulate gene expression, thus controlling the cell growth, differentiation, and proliferation. ERK pathway also controls the proliferation and differentiation and survival of cells. DAP prevented the RCC proliferation by inhibiting ERK/MAPK pathway and upregulated the differentiation mediated by p38 MAP kinase. It also suppressed the G_1_ to S phase transition by inhibiting DNA synthesis at 10 and 50 µM in AQ-498 cells (Finn et al., 2004). The anticancer mechanism of DAP is shown in Figure 4. p38 MAP kinase is intrinsically involved in mediating the effect of DAP in A-498 cells. Moreover, DAP is involved in promoting the cellular maturation and is considered to be a new less toxic approach for treating poorly differentiated RCC (Finn et al., 2004).

#### 3.12.2 Ovarian cancer

Ovarian cancer is the sixth most common cancer among European women (Colombo et al., 2006). Autophagy, apoptosis, and ROS production can trigger cell death and help to treat cancer. AMPK/Akt/mTOR pathway is associated with autophagy and apoptosis. In an earlier study, DAP exhibited the anticancer potential in A2780 xenograft tumor model against ovarian cancer at 0, 5, 10, 20, and 40 μg/ml *in-vitro* and 30 mg/kg *in-vivo* by inducing cell death, increasing ROS production, inducing autophagy, and inhibiting the cell proliferation (Fan et al., 2021a).

#### 3.12.3 Leukemia

Benzene is a chemical present in the atmosphere that can cause different types of leukemia. Exposure to the vapors of benzene leads to oxidative damage, inflammatory responses, changes in cell cycle progression, and DNA damage (Huff, 2007). In a benzene-induced leukemia study, treatment of rats with DAP at 12.5, 25, and 50 mg/kg caused an increased blood count, and hemoglobin concentration, reduced the level of inflammatory mediators, and inhibited ROS production to retard cancer progression (Pei et al., 2021).

#### 3.12.4 Liver cancer

A previous study reported the therapeutic potential of DAP against liver cancer. Hepatocellular carcinoma (HCC) was induced in Wistar rats by diethyl nitrosamine (DEN) (200 mg/kg) and its effect was enhanced by phenobarbital for 4 weeks. DAP (at 10, 20, and 30 mg/kg) repressed the biochemical parameters with enhanced levels of GSH, glutathione S-transferase (GST), SOD and CAT while decreasing the level of MDA. It also reduced the inflammatory markers such as COX-2, NF-κB, prostaglandin (PGE2), IL-1β, IL-6, and TNF-α in treated rats (Li et al., 2022).

In another study, DAP inhibited the progression of hepatocellular carcinoma in Huh7 and SK-HEP-1 cell lines. DAP suppressed the cell viability and tumorigenesis, promoted the apoptosis of cells, and induced the arrest the cells in G1 phase dose-dependently which were rescued by SKL 2001, an activator of Wnt/β-catenin signaling. Thus, DAP exerted an antitumor role through the inactivation of Wnt/β-catenin signaling (Liu et al., 2022).

#### 3.12.5 Breast cancer

Cell proliferation and estrogenicity lead to the tumor development in breast. DAP acts at different phases of cell cycle thus controlling cell proliferation and tumor development. Cyclin D1 is a major protein for the initiation of cell cycle and proliferation of cells. In a previous study, DAP suppressed cyclin D1, thereby preventing the proliferation in MCF-7 cells. It is also a protein kinase inhibitor which leads to the inhibition of proliferation. It did not possess estrogenic activity (Jiménez-Orozco et al., 2011).

In another study. DAP inhibited p-AKT which reduced NF- κB in mammary cancer. It was considered to be an effective agent in the treatment of mammary cancer in rats by suppressing the Nrf-2-Keap_1_ pathway and NF- κB expression (Kumar et al., 2016a).

### 3.13 Nephroprotective action

The DAP showed nephroprotective effect against cisplatin-induced nephrotoxicity by suppressing the NF-κB signaling pathway and activating the Nrf2 pathway when C57BL/6 mice were treated with DAP at 2.5–10 mg/kg. It decreased the blood urea nitrogen and creatinine levels along with the reduction of ROS (Zhang et al., 2018). Another study stated that the DAP (at 40 mg/kg) restored the weight loss, blood urea, kidney index, and creatinine levels in cisplatin-induced acute nephrotoxicity. It remarkably increased sirtuins (SIRT1, SIRT6) and Nrf2 with an increased SOD and GSH levels, and the reduction in MDA and MPO levels in wild-type mice (Fan et al., 2020). The nephroprotective mechanism of DAP is shown in Figure 4. A study also reported the preventive potential of DAP in diabetic nephropathy in mesangial cells at 10-40 µM by preventing cell proliferation, protection against oxidative stress and inflammation by targeting Nrf2/keap1, and Akt/NF-kB inflammatory pathways (Shen et al., 2017; Xu et al., 2019).

DAP protected the mice from gentamicin-induced nephrotoxicity at 40 mg/kg by preventing renal injury and decreasing cell damage. It upregulated the expression of Nrf2, and antioxidant enzymes such as HO-1, NQ0_1_, GCLC and GCLM (Fan et al., 2021b).

### 3.14 Other actions of Daphnetin

Song et al. investigated the potential effect of DAP as immunosuppressive agent in BALB/c mice using a 100 μl emulsion comprising of 100 μg OVA as prototype antigen. DAP (at 5, 10, and 20 mg/kg i.p.) downregulated the OVA-specific antibody IgG subclasses IgG1 and IgG2b and reduced the growth of Th1 and Th2 cytokines as well as restrained *in-vivo* splenocytes proliferation (Song et al., 2021).

It was reported that the pretreatment with DAP (at 1, 10, 20, and 40 µM) improved the cell viability in rat insulinoma (INS-1) cells that were previously exposed to streptozotocin (STZ) as compared to INS-1 cells (Negative control). It also improved the insulin secretion in the INS-1 cells. Thus, the antidiabetic effect of DAP relied on insulin stimulating, and antiapoptotic actions (Vinayagam and Xu, 2017).

DAP (at 4–16 mg/kg) considerably improved the experimental colitis by suppressing the colonic inflammation, improving colonic integrity, and restoring the immune and metabolic homeostasis. It increased the abundance of short-chain fatty acid producing microbiota of gut that were responsible for the increased development of T _reg_ cells and the reduced pro-inflammatory T_h_ 17 cell differentiation (Ji et al., 2019).

Previously, it was found that the DAP inhibited melanin biosynthesis by suppressing the expression of microphthalmia associated transcription factor responsible for melanogenesis, and also inhibited melanogenic enzymes such as tyrosine and tyrosine-related proteins in B16F10 cells. DAP downregulated the phosphorylation of kinases such as PKA, ERK, mitogen and stress activated protein kinase (MSK)-1 and cAMP response element binding protein (CREB). It inhibited the melanin synthesis, and exhibited the anti-pigmentation activity by modulating PKA/CREB, and ERK/MSK1/CREB pathways (Nam et al., 2022).

DAP possesses analgesic action. In a previous study, DAP at 10 mg/kg averted reserpine induced fibromyalgia (chronic pain syndrome along with depression) in mice. DAP effectively averted fibromyalgia by downregulating monoamine oxidase-A (MAO-A), glutamate level, IL-1β, and TNF-α while elevating the GSH, dopamine, serotonin and norepinephrine levels (Singh et al., 2021b).

A previous study reported that DAP reduced Toll-like receptor-4 (TLR4) expression and suppressed the activation of the NF-κB signaling pathway in acute pancreatitis showing its potential to avert pancreatitis (Liu et al., 2016b). DAP as an emulsion [locus bean gum (0.5%) and sodium alginate (1.5%)] is used in the food industry as an additive, preservative and packaging material. It is used as a preservative because of antimicrobial and antioxidant properties (Liu et al., 2021; Cheng, 2022).

Wen et al. investigated the percutaneous absorption of DAP in rat abdominal skin using various chemical enhancers. The experiment was performed using isopropyl myristate as a vehicle while other enhances of *O*-acylmenthol derivatives were synthesized from which only M-LA was explored to improve the DAP permeation. Its effects were also pronounced when DAP was used with span 80 (Wen et al., 2009).

Yang and his coworkers investigated the effect of DAP on ischemia repurfusion (I/R) injury. DAP (at 2.5, 5,10, and 20 mg/kg) decreased the myocardial I/R injury with improved cardiac function in treated cells. It reduced the apoptosis, oxidative stress, and inflammation both under *in-vitro* and *in-vivo* experiments. It also reduced the risk of ventricular arrhythmias by downregulation of TLR4, MyD88, and NF-κβ in I/R mice (Yang et al., 2021b).

In another study, DAP exhibited anti-angiogenic properties through inhibition of different stages of angiogenesis such as migration, invasion, and tube formation. It suppressed the NF-κB pathway, TNF-α induced IκBα degradation and translocation of the NF- κB-p65 protein. It significantly decreased the expression of c-Src, FAK, ERK1/2, Akt, VEGFR2, Inos, and MMP2 as well as induced apoptosis (Kumar et al., 2016b).

DAP also exhibited the protection against LPS induced inflammatory bone destruction in murine osteolysis model. It inhibited RANKL-induced osteoclast differentiation, fusion and bone resorption. It inhibited the activation of ERK and NFAT_c_1 signaling cascade, so it has the potential to be used for the treatment of inflammatory osteolysis (Wu et al., 2019). Zhou and Zhang et al. investigated the *in-vitro* integration of DAP-Cu (II) complex with calf thymus DNA (ctDNA). The findings exhibited that the DAP and Cu^2+^ exerted synergistic pharmacological actions (Zhou et al., 2016).

## 4 Toxicity studies

A previous study reported the Minimum inhibitory concentration (MIC of DAP (25 μg/ml) against highly resistant *H. pylori* isolated from human gastric antrum. Using the Cell Counting Kit-8 (CCK-8), the sub-minimum inhibitory concentration (MIC) of DAP was studied in GES-1 cells. DAP was well tolerated by GES-1 cells, and there was insignificant difference on cytotoxic effect of DAP by culture media conditions (Wang et al., 2019). Furthermore, the acute toxicity of DAP was evaluated using the Bacterial reverse mutation assay (Ames test), which revealed no genetic toxicity. Bone marrow micronucleus test revealed that DAP) had no effect on mouse bone marrow cells at various concentrations (0.75, 1.5, and 3 mg/kg). DAP was prepared for an acute skin allergy test at a final concentration of 4 mg/ml, indicating that it is non-allergenic. A local mucosa stimulation test was done on the oral mucosa of rabbits which revealed no oral mucosa ulceration, erosion, erythema and irritation caused by DAP.

In mice, the maximal oral toxic dose of DAP was greater than 100 mg/kg (Nanzhen et al., 2018). Hippocampal HT-22 cell line was used with 5 mM glutamate and different concentrations of DAP. After 12 h of incubation, 100 mΜ DAP protected HT-22 cells in concentration dependent manner against glutamate toxicity (Du et al., 2014). BV2 microglia were used and treated with 0-160 μM concentrations of DAP which showed insignificant change in cell survival rate (Yu et al., 2014a). Indeed, the *in-vitro* and *in-vivo* studies confirmed that DAP was devoid of any significant toxicity at pharmacologically relevant concentrations.

## 5 Structure activity relationship

There is a series of DAP derivatives ranging from 1-22 that showed moderate inhibitory or activating action on GPCRs resultantly responsible for copious pharmacological activities. The activity of GPCRs depends on the chemical alteration of hydroxyl groups at the C-7, C-8, and C-3/C-4 positions of DAP (Satô and Hasegawa, 1969; Wang et al., 2020a). The C-7 and C-8 substituents were generated through phenolic O-acylation/O-alkylation (nucleophilic acyl/alkyl substitution), whereas the C-3 and C-4 substituents were formed using Pechmann condensation. DAP 2, 3, 4, 5, 15, 16, 18, 19, and 20 cause moderate activation on GPCRs while 3-5, and 19 exhibit profound activation with EC50 of 1.18–1.91 nM (Wang et al., 2020a).

Substitution at C-3 or C-4 of DAP produces different derivatives. The anti-oxidant activities of different DAP derivatives were evaluated. The catechol group was considered a key pharmacophore for the anti-oxidant activity. The introduction of electron-withdrawing hydrophilic group at the C-4 position increased the anti-oxidant potential but it was not observed with C-3 substitution. Introduction of the hydrophobic phenyl group produced negative effect on the anti-oxidant activity at C-3 and C-4. The 4-carboxymethyl DAP exerted the most powerful anti-oxidant activity. It also displayed strong metabolic stability (Dar et al., 2015; Xia et al., 2018b).

## 6 Discussion

The inclusion of multiple research investigations on pharmacological mechanisms of DAP in treating numerous chronic conditions was a main emphasis of the current study. Consequently, the review offers a comprehensive outline of the prospective medicinal uses of this phytochemical. The current study uncovered the pharmacological and therapeutic benefits of DAP for human health. Several medicinal plants are rich source of bioactive compounds which exhibit numerous pharmacological activities, minimal side effects, and the potential source of novel drugs for the treatment of diseases. Many drugs currently available in the market have either been directly or indirectly derived from the traditional plants. By reviewing the available information, it was determined that DAP was a bioactive constituent with a variety of effects against bacterial microorganisms, inflammation, malarial parasite, viral infections, cardiovascular diseases, rheumatoid arthritis, kidney disorders, cerebral disorders, various cancers, lung infections, melanogenesis, bowel diseases, oxidative stress, diabetes mellitus, and others. However, its impact against different cancer types are particularly important in the therapy of numerous malignant diseases.

DAP is a simple coumarin derivative with a variety of therapeutic benefits in preclinical research and mostly isolated from the *Daphne* genus*.* Thus, there are areas to work on the isolation from other genus and synthesis in laboratory. Furthermore, identification of various intermediate metabolites may broaden the range of biologically active compounds to be tested for various ailments.

In numerous preclinical researches, DAP’s neuroprotective effects have been thoroughly documented. Studies have demonstrated the protective effect of DAP against ischemic/reperfusion injury, and spatial memory impairment caused by CUS, NMDA-induced excitotoxicity, glutamate-excited HT-22 cells, as well as cerebral ischemia (Liao et al., 2013; Du et al., 2014; Yang et al., 2014; Liu et al., 2016a; Berman and Bayati, 2018). The neuroprotective effect can be significantly achieved by the modification of the TLR-4/NF-κB, HSP70, JAK/STAT, and Nrf-2/HO^−1^ downstream pathways (Figure 4). DAP’s potential as a neuroprotective compound could further be supported in preclinical research by examining its impact on the Aβ amyloid, tau, Parkinson’s, and Huntingtin proteins.

In cancer, cells divide uncontrollably and metastasize other tissues. The findings of current review indicated that the DAP was pharmacologically effective against different type of cancers including cancers of kidney, liver, ovary and leukemia via inhibiting the proliferation and promotion of apoptosis making it a viable adjuvant in the treatment of cancer.

The inflammation and oxidative stress are increased in severe joint inflammatory conditions such as osteoarthritis and RA. The upsurge of pro-inflammatory cytokines, NF-κB, myeloperoxidase, iNOS, NOS, COX-2, and other mediators worsen the disease (Prasad et al., 2021). Thus, the analysis of previous studies indicated that the DAP was effective in retarding the progression of RA and other inflammatory diseases even at low doses via inhibiting the pro-inflammatory cytokines, NF-κB and MMP levels, and restored the protein expressions.

The DAP and derivatives were effective against several bacterial infections as well as different malarial parasites *via* suppressing the metabolic functions of these microbes as demonstrated by diverse *in-vitro* and *in-vivo* studies. Such studies are need to be extended to antibiotic resistant microbes so as to treat and prevent drug resistant infections. The hepatoxicity could be due to infections, malignant diseases and drug therapy. Previous investigations indicated that the DAP had antioxidant potential and inhibited lipid and protein oxidation, decreased ROS, and pro-inflammatory cytokines to prevent and treat hepatotoxicity (Khan et al., 2019).

Several intriguing pharmacological investigations on DAP against different diseases have outlined the mechanisms supporting the use of DAP as supplementary therapy. Further research is needed on long-term toxicity, information on potential medication interactions and its effect as adjuvant therapy against chronic diseases. To further support the clinical value in medical practice, supplementary clinical trials are also required. Previous studies show that DAP is a suitable candidate for the drug development.

## 7 Conclusion and future perspectives

This review provides pertinent information regarding the pharmacological aspects of DAP to explore its hidden potential as it targets various molecular and cellular pathways to combat numerous inflammatory disorders, infectious diseases, neurological disorders, hepatotoxicity, nephrotoxicity, psoriasis, diabetic nephropathy, leukemia, and other cancers. DAP exhibited no mutagenic effect, allergenic action, sensitization, mucosal irritation, erythema, and mortality in toxicity studies. The information summarized above will be used for the development of an effective formulation for the treatment of various ailments without significant adverse/toxic effects.

On the basis of literature reviewed, it has been found that DAP exhibited remarkable pharmacological profile and it could be used as a treatment or adjuvant for the treatment of different disorders. Thus, still its biosynthesis and structure activity relationship should be critically analyzed. The effect of DAP on *H. pylori* was investigated but other microorganisms causing gastrointestinal infections should be investigated to reduce their impact on human and animal health. The development of nanoformulations of DAP need attention to enhance its therapeutic effect and half-life. The synergistic effect of DAP with commercially available drugs should be studied to enhancing their effects in treating various diseases. As DAP attenuates the activation of microglia that plays a crucial role in the pathogenesis of multiple neurodegenerative diseases, this evidence suggests the possibility of DAP as treatment option for Huntington and Parkinson’s disease. Further research should be implicated to explore its various bioactivities and mechanisms. Toxicity study of DAP and its derivatives should be conducted in detail to assure their safety in human and animals (Lu et al., 2021).
